# Fulminant pheochromocytoma crisis triggered by glucocorticoids: ultra-early adrenalectomy under ECMELLA support

**DOI:** 10.3389/fendo.2026.1889987

**Published:** 2026-08-26

**Authors:** Ludovico Di Gioia, Emanuele Rollo, Mariangela Caporusso, Lucrezia Matera, Giovanna Magnesa, Marco Vulpi, Giuseppe Ingravallo, Salvatore Maurizio Maggiore, Massimo Grimaldi, Tomaso Bottio, Pasquale Ditonno, Vito Marco Ranieri, Francesco Giorgino, Sebastio Perrini

**Affiliations:** 1 Section of Endocrinology, Ecclesiastical Entity Regional General Hospital “Francesco Miulli”, Bari, Italy; 2 Department of Medicine and Surgery, Section of Endocrinology, Libera Università Mediterranea (LUM), Bari, Italy; 3 Department of Precision and Regenerative Medicine and Ionian Area, Section of Anesthesiology and Intensive Care, University of Bari Aldo Moro, Bari, Italy; 4 Section of Anesthesiology and Intensive Care Medicine, Ecclesiastical Entity Regional General Hospital “Francesco Miulli”, Bari, Italy; 5 Department of Medicine and Surgery, Section of Anesthesiology and Intensive Care Medicine, Libera Università Mediterranea (LUM), Bari, Italy; 6 Department of Precision and Regenerative Medicine and Ionian Area, Section of Urology, Andrology and Kidney Transplantation, University of Bari Aldo Moro, Bari, Italy; 7 Department of Precision and Regenerative Medicine and Ionian Area, Section of Pathology, University of Bari Aldo Moro, Bari, Italy; 8 Department of Medicine and Surgery, Section of Cardiology, Libera Università Mediterranea (LUM), Casamassima, Bari, Italy; 9 Department of Precision and Regenerative Medicine and Ionian Area, Cardiac Surgery Unit, University of Bari Aldo Moro, Bari, Italy; 10 Department of Precision and Regenerative Medicine and Ionian Area, Section of Internal Medicine, Endocrinology, Andrology and Metabolic Diseases, University of Bari Aldo Moro, Bari, Italy

**Keywords:** adrenalectomy, case report, catecholamine-induced cardiomyopathy, ECMELLA, mechanical circulatory support, pheochromocytoma, steroids

## Abstract

Pheochromocytoma crisis is a rare but life-threatening condition that may present with refractory cardiogenic shock and malignant arrhythmias. In this setting, the patient is often too unstable for surgery, although tumor removal remains the only definitive treatment. We report the case of a 57-year-old woman with an adrenal mass who developed a fulminant catecholaminergic crisis after high-dose glucocorticoid administration for contrast allergy prophylaxis. She presented with severe metabolic acidosis (pH 6.98, lactate 14.8 mmol/L), flash pulmonary edema, and acute catecholamine-induced cardiomyopathy with a Takotsubo pattern (LVEF 25%). Initial support with Impella CP was insufficient due to electrical storm and hypermetabolic state. Mechanical support was escalated to combined veno-arterial extracorporeal membrane oxygenation (VA-ECMO) and Impella (ECMELLA). Because of persistent instability, ultra-early robot-assisted adrenalectomy was performed under full mechanical support. The patient was successfully weaned from Impella on postoperative day (POD) 1 and from ECMO on POD5, with progressive cardiac recovery (LVEF 35% at POD14). To our knowledge, this is one of the first (or the first) reported cases of robot-assisted adrenalectomy performed under full ECMELLA support without prior durable hemodynamic stabilization, suggesting a potential role for this strategy in highly selected patients with refractory pheochromocytoma crisis. It also underscores glucocorticoids as a critical and underrecognized trigger.

## 1 Introduction

**Figure 1:**
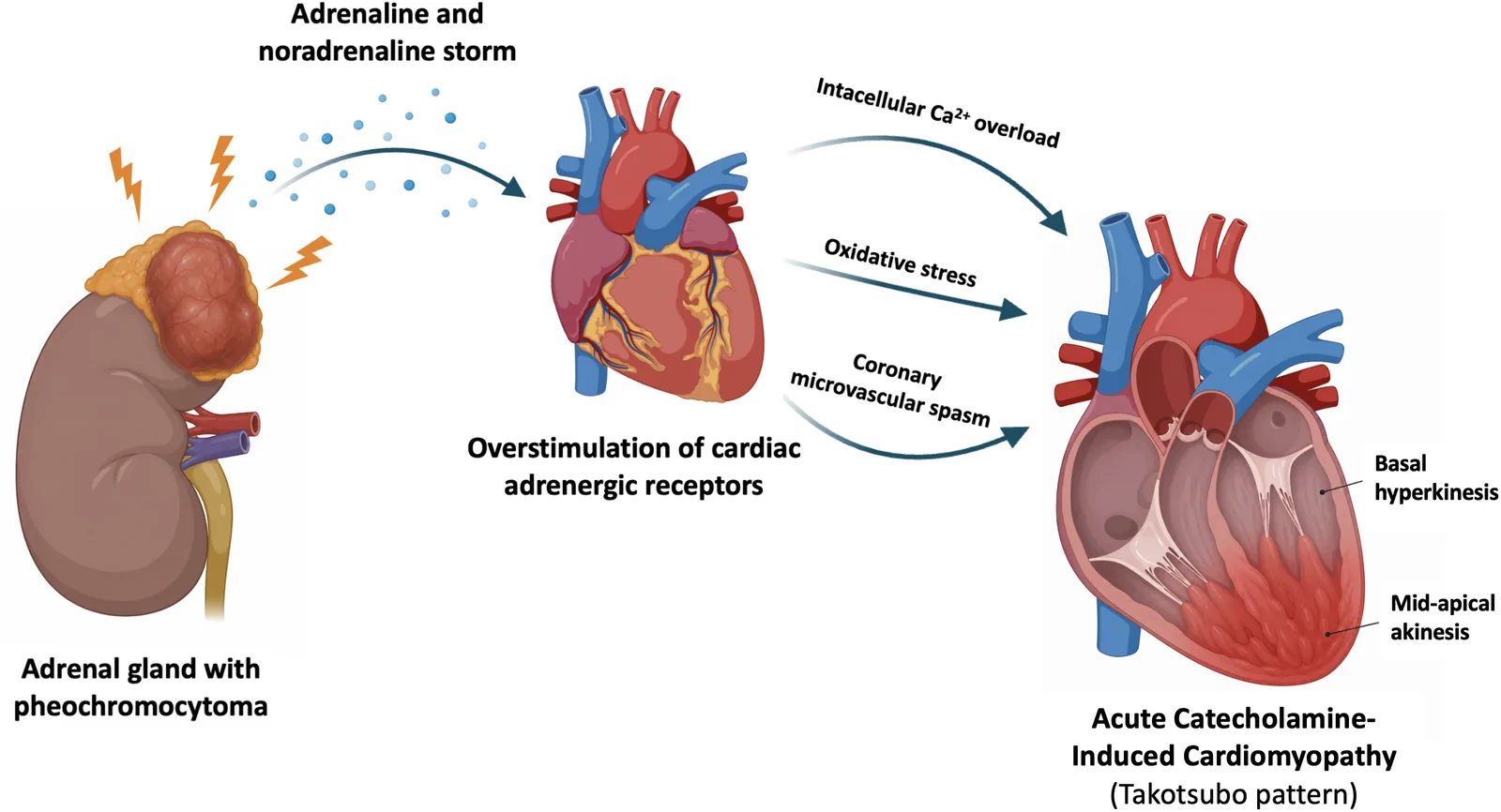
Pathophysiology of acute catecholamine-induced cardiomyopathy (ACIC) in pheochromocytoma. Schematic illustration demonstrating the mechanism of ACIC. The pheochromocytoma releases a massive surge of catecholamines (epinephrine/norepinephrine), following a triggering stimulus (represented in the figure by lightning bolts), leading to supraphysiological stimulation of cardiac adrenergic receptors. This results in intracellular calcium overload, oxidative stress, and severe coronary microvascular spasm, ultimately causing myocardial stunning and the classic apical Takotsubo pattern observed in this case, characterized by mid-apical akinesis and basal hyperkinesis. Created in BioRender. https://BioRender.com/413uen5.

**Figure 2:**
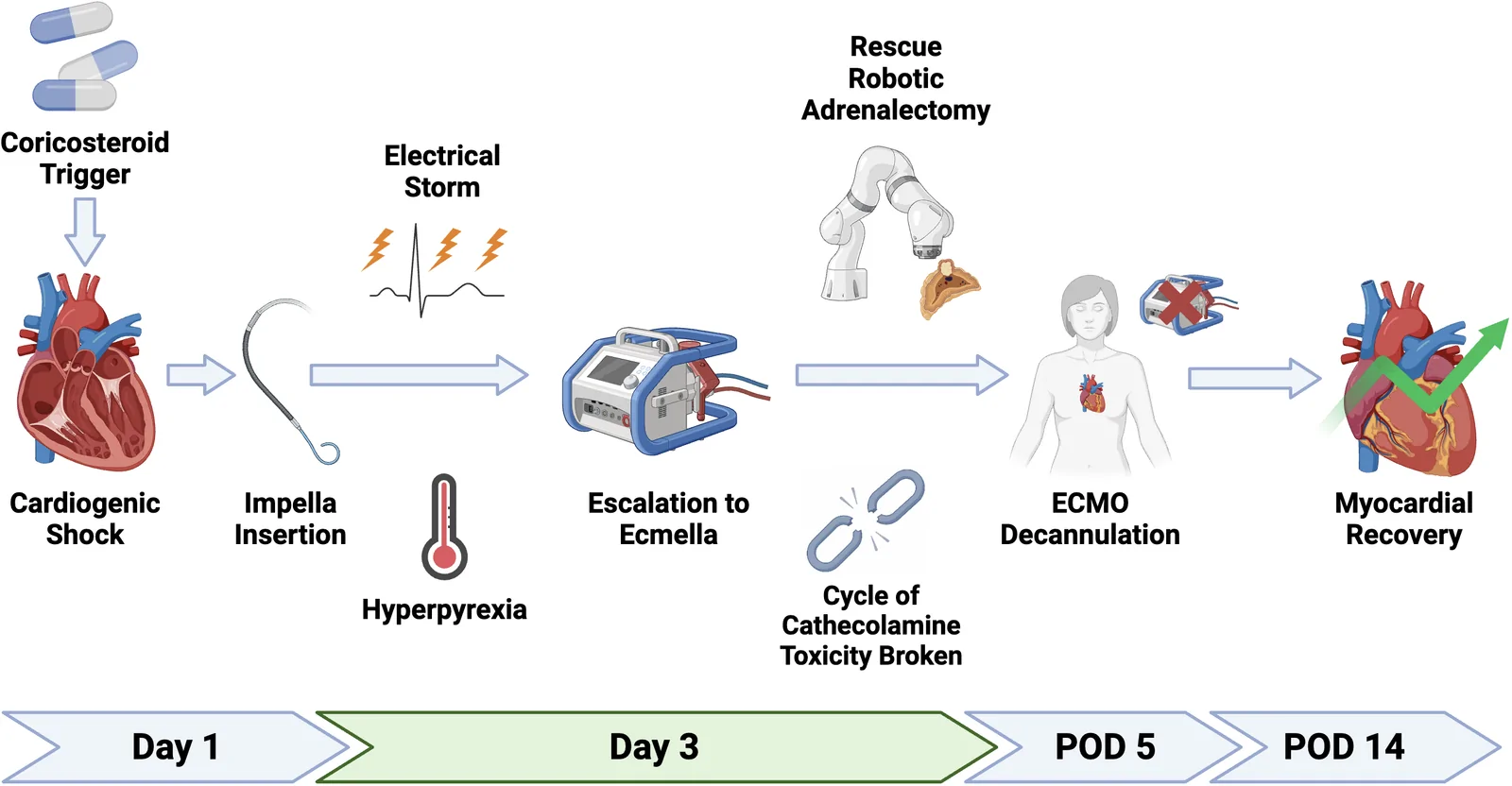
Strategic timeline highlighting ultra-early source control. Timeline of major clinical events illustrating the rapidity of the intervention. Day 1: Corticosteroid trigger leads to cardiogenic shock (pH 6.98) and Impella insertion. Day 3: Onset of electrical storm and hyperpyrexia prompts escalation to ECMELLA. Crucially, Rescue Robotic Adrenalectomy is performed just hours later on the same night, breaking the cycle of catecholamine toxicity. POD 5: ECMO decannulation. POD 14: Evidence of myocardial recovery trend. Created in BioRender. https://BioRender.com/413uen5.

Pheochromocytoma is a neuroendocrine tumor of the adrenal medulla with a highly variable clinical presentation. While classically associated with paroxysmal hypertension and adrenergic symptoms, it may be clinically silent or incidentally detected while still retaining the potential to trigger life-threatening cardiovascular complications. Among the most dramatic manifestations is acute catecholamine-induced cardiomyopathy (ACIC) (**Figure 1**), a syndrome driven by direct myocardial toxicity, microvascular dysfunction, intracellular calcium overload, and coronary vasospasm. Clinically, ACIC may mimic acute coronary syndrome or present with Takotsubo or reverse Takotsubo patterns (1). Management of ACIC related to pheochromocytoma is traditionally biphasic, consisting of initial medical stabilization with alpha-adrenergic blockade, with or without mechanical circulatory support (MCS), followed by delayed adrenalectomy after cardiac recovery (2). However, a subset of patients develops a fulminant course characterized by refractory electrical storm, hemodynamic collapse, and hypermetabolic failure. In these rapidly progressive scenarios with multiorgan failure, pharmacological stabilization may be ineffective and prolonged MCS exposure carries substantial risk, including bleeding, infection, and vascular complications (3). Here, we describe administration of glucocorticoid-triggered pheochromocytoma crisis with fulminant ACIC, managed by sequential escalation to combined Impella and veno-arterial extracorporeal membrane oxygenation (VA-ECMO) (ECMELLA) support. Notably, the ECMELLA strategy enabled an unprecedented ultra-early robotic adrenalectomy performed under full biventricular mechanical circulatory support, allowing definitive source control despite otherwise refractory hemodynamic instability (**Figure 2**).

## 2 Case presentation

**Figure 3:**
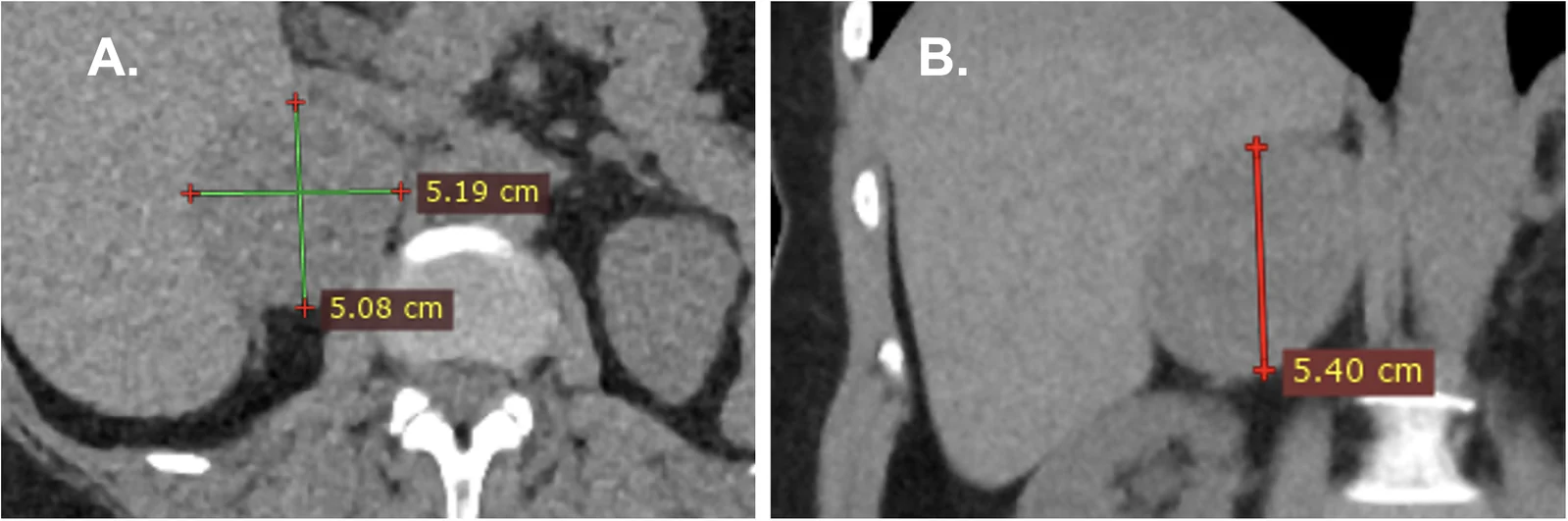
Preoperative computed tomography (CT). Axial **(A)** and coronal **(B)** CT images demonstrating a heterogeneous 5 cm mass in the right adrenal gland, consistent with a pheochromocytoma, prior to the onset of the adrenergic crisis.

A 57-year-old postmenopausal woman underwent a non-contrast abdominal computed tomography (CT) scan on 25 October 2025 for right flank pain. The scan revealed an incidental right adrenal mass, described as a rounded lesion measuring approximately 50 mm in maximum diameter, located in the adrenal lodge, with homogeneous attenuation of 33 Hounsfield Units (HU) on unenhanced acquisition. Given the lesion size (>4 cm) and density (>20 HU) (**Figure 3**), the radiologist explicitly recommended further characterization with contrast-enhanced magnetic resonance imaging (MRI). However, MRI could not be performed due to the presence of a metallic orthopedic prosthesis in the forearm, implanted several years earlier following traumatic injury, which was considered a contraindication to MRI. Approximately one month later (25 November 2025), the patient underwent endocrinological evaluation. At that time, apart from the initial right flank pain, the patient did not report any other symptoms suggestive of catecholamine excess, including headache, palpitations, diaphoresis, or paroxysmal hypertension. However, she appeared noticeably anxious in relation to the recent discovery of the adrenal mass. Blood pressure and heart rate were well controlled under antihypertensive therapy (olmesartan medoxomil/amlodipine 20/5 mg once daily and bisoprolol 2.5 mg once daily), which the patient had been taking for several years. Vital signs were stable, with blood pressure within the normal range and heart rate in the mid-80s bpm. Following the endocrinological assessment, the patient was referred for hospital admission to complete the diagnostic work-up and staging of the adrenal lesion according to current guideline recommendations (4). To avoid interference with hormonal evaluation, beta-blockade therapy was discontinued and replaced with verapamil 120 mg twice daily.

Baseline biochemical and imaging assessments were initiated in accordance with international guidelines for the evaluation of adrenal incidentalomas. Investigations included serum cortisol and adrenocorticotropic hormone (ACTH) with circadian rhythm assessment, plasma aldosterone and renin, and a 24-hour urine collection for urinary free cortisol and urinary metanephrines (**Table 1**). A contrast-enhanced CT scan of the chest and abdomen was scheduled for December 1. At that time, results of urinary metanephrines were still pending.

**Table 1:** Baseline biochemical evaluation.

Parameter	29/11/25	03/12/25	Reference
Normetanephrine (µg/24 h)	693	13, 153	52-341
Metanephrine (µg/24 h)	13, 235	71, 574	88-444
3-Methoxytyramine (µg/24 h)	234	417	0-1, 000
Urinary free cortisol (µg/24 h)	41.8	–	4.2-176
ACTH (pg/mL)	24.4	–	6-48
Serum cortisol, 8 a.m. (µg/dL)	7.8	–	3.7-19.4
Serum cortisol, 12 p.m. (µg/dL)	4.1	–	–
Aldosterone (pg/mL)	70.3	–	17.6-232
Renin (µIU/mL)	12.7	–	2.7-39.8
Chromogranin A (nmol/L)	8.8	–	0.0-3.0
Neuron-specific enolase (ng/mL)	5.3	–	<11.1

ACTH, adrenocorticotropic hormone.

### 2.1 Triggering event and early clinical deterioration

Steroid premedication was administered before contrast-enhanced imaging because of a history of allergic reactions, consisting of prednisone 50 mg scheduled at 13, 7, and 1 hour before the scan, plus intramuscular chlorphenamine maleate 10 mg/1 mL immediately prior to imaging. Shortly after the second prednisone dose, the patient developed new-onset arterial hypertension (160/90 mmHg), without associated symptoms. Intravenous furosemide 20 mg was administered, with no significant blood pressure response. Over the following hours, blood pressure rapidly escalated to 220/110 mmHg, and was accompanied by headache, tremor, flushing, and palpitations, consistent with an acute catecholaminergic surge. A pheochromocytoma crisis was clinically suspected. Empirical treatment with oral doxazosin failed to control blood pressure. In the absence of response and given the rapid clinical deterioration, the resuscitation team was promptly activated and continuous intravenous alpha-adrenergic blockade with urapidil was initiated. An electrocardiogram showed sinus tachycardia (127 bpm) with premature supraventricular complexes and inferolateral myocardial infarction pattern. The patient was transferred to the intensive care unit (ICU) and intubated shortly thereafter. Despite initial partial hemodynamic response to urapidil, laboratory findings rapidly worsened, with severe hyperglycemia, acute kidney injury, and marked elevation of cardiac biomarkers (**Table 2a**). Within hours, the clinical scenario evolved into overt metabolic failure, with arterial blood gas analysis showing acidemia (pH 7.34) and hyperlactatemia (11.3 mmol/L) (**Table 2a**). Cardiology reassessment confirmed persistent tachycardia (~130 bpm) and rising troponin levels.

**Table 2a:** Temporal evolution of arterial blood gas parameters and laboratory findings during pheochromocytoma crisis and mechanical circulatory support.

Parameter	01/12 h 12:27	01/12 h 14:45	01/12 h 18:08	03/12 h 00:25	04/12 PRE- ECMELLA	04/12 POST- ECMELLA*	05/12	15/12
pH	6.98	–	7.34	7.49	7.39	7.53	7.50	7.46
PaO_2_ (mmHg)	57	–	96	121	77	112	138	87
PaCO_2_ (mmHg)	33	–	39	46	45	34	42	31
SO_2_ (%)	79.9	–	99	99.7	97	99	97	98
FiO_2_ (%)	26	–	100	50	50	80	60	28
P/F ratio	219	–	96	242	154	140	230	311
Lactate (mmoL/L)	14.8	–	11.3	2.7	2.9	2.2	1.4	0.8
HCO_3_^-^ (mmoL/L)	7.8	–	21	35.1	27.2	28.4	33	22
Glucose (mg/dL)	>750	699	235	158	176	166	135	97
Creatinine (mg/dL)	–	1.91	2.06	1.70	2.37	2.39	1.75	0.80
AST (U/L)	–	41	282	1, 451	2, 109	1, 909	884	65
ALT (U/L)	–	16	127	1, 183	1, 828	1, 770	1, 059	102
LDH (U/L)	200	273	776	2, 567	–	–	–	–
Myoglobin (ng/mL)	43	5, 898.8	>1, 200	–	–	–	–	–
CK (U/L)	150	473	1, 407	2, 274	–	–	–	–
Troponin (pg/mL)	7.8	9, 725	>50, 000	59, 295.7	40, 853.8	12, 546.8	6, 884.9	308.4
NT-proBNP (pg/mL)	–	–	–	–	–	–	11, 472	10, 243
D-dimers (ng/mL)	–	3, 302	–	13, 687	–	–	–	–

*POST-ECMELLA refers to values measured after ECMELLA initiation.

pH, potential of hydrogen; PaO_2_, partial pressure of oxygen; PaCO_2_, partial pressure of carbon dioxide; SO_2_, oxygen saturation; FiO_2_, fraction of inspired oxygen; P/F ratio, ratio of arterial oxygen partial pressure to fraction of inspired oxygen (PaO_2_/FiO_2_); HCO_3_^–^, bicarbonate; AST, aspartate aminotransferase; ALT, alanine aminotransferase; LDH, lactate dehydrogenase; CK, creatine kinase; NT-proBNP, N-terminal pro–B-type natriuretic peptide.

Electrocardiographic abnormalities evolved with resolution of diffuse ST-segment elevation and development of QS complexes in the right precordial leads. Intravenous labetalol provided only transient blood pressure control and metoprolol infusion was initiated. Transthoracic echocardiography revealed severe left ventricle systolic dysfunction (left ventricle estimated ejection fraction, LVEF, 25–30%) (**Table 2b**), with mid-apical akinesis and basal hyperkinesis, consistent with a classic apical Takotsubo pattern. Right heart chambers were normal in size, with no significant valvular disease. A mild pericardial effusion was noted. A bedside chest X-ray imaging showed interstitial pulmonary edema with bilateral pleural effusions. During the night, blood pressure became labile with episodes of excessive hypotension under continuous intravenous alpha-blockade, requiring transition to oral therapy. On December 2, endocrinology reassessment confirmed a pheochromocytoma complicated by hypertensive crisis, acute heart failure with interstitial pulmonary edema, and hepatic cytolysis. The patient remained intubated and sedated, receiving oral doxazosin and intravenous beta-blockade. Hemodynamic parameters showed only transient stabilization (blood pressure 80–120/40–80 mmHg, heart rate 110–120 bpm) with partial recovery of urine output. Chromogranin A was elevated, while urinary metanephrines were still pending. At this stage, despite combined pharmacological therapy, the clinical course remained unstable, with ongoing evidence of catecholamine-driven cardiovascular and metabolic deterioration following glucocorticoid exposure.

**Table 2b:** Temporal evolution of LVEF during pheochromocytoma crisis, mechanical circulatory support, and after cardiac rehabilitation.

Parameter	01/12	02/12	02/12	05/12	10/12	12/12	19/12	22/12	09/01
LVEF (%)	25-30	25	25	25	25	30	35	40	60

LVEF, left ventricle estimated ejection fraction.

### 2.2 Refractory cardiogenic shock and initiation of Impella support

Repeat transthoracic echocardiography confirmed severe left ventricular systolic dysfunction (LVEF approximately 25%) with Takotsubo pattern, characterized by mid-apical akinesis and basal hyperkinesis (1). Right ventricular function was mildly impaired, and a small, hemodynamically insignificant pericardial effusion was present. In the setting of refractory cardiogenic shock and severe left ventricular (LV) dysfunction, mechanical circulatory support was initiated. Coronary angiography excluded obstructive coronary artery disease, and a percutaneous microaxial flow pump (Impella CP^®^, AbioMed) was implanted to provide active left ventricular unloading.

Despite initial support, the clinical course rapidly deteriorated. During the night, the patient developed extreme hemodynamic instability, with abrupt fluctuations between severe hypotension and hypertensive surges, associated with alternating tachyarrhythmia and bradyarrhythmia. Reassessment the following morning confirmed persistent severe left ventricular systolic dysfunction (LVEF ~25%), along with ongoing ECG abnormalities, including persistent ST-segment elevation and Q waves in the anterior–lateral leads.

Abdominal ultrasonography demonstrated increased intralesional heterogeneity with internal fluid components, raising concern for acute degenerative or hemorrhagic tumor changes potentially contributing to the marked hemodynamic instability due to ongoing catecholamine release. The clinical course was further complicated by recurrent ventricular tachycardia requiring intravenous lidocaine. An intensified hemodynamic control strategy was attempted using continuous intravenous alpha-blockade (urapidil) combined with short-acting beta-blockade (landiolol) (5). However, treatment was poorly tolerated, as urapidil repeatedly induced profound hypotension despite careful titration and volume support.

At this stage, the patient remained in a catecholamine-driven shock state, with extreme blood pressure variability and malignant arrhythmias. The clinical course had already been marked by profound metabolic failure, with a nadir pH of 6.98 and a peak lactate concentration of 14.8 mmol/L, indicating severe systemic hypoperfusion. Although these parameters subsequently improved under intensive treatment and Impella support, extreme hemodynamic variability, recurrent arrhythmias, and progressive organ dysfunction persisted.

Despite maximal medical therapy and Impella support, durable hemodynamic stabilization could not be achieved. Mechanical support alone was insufficient to control the underlying disease process. Given the ongoing deterioration, urgent adrenalectomy under extracorporeal membrane oxygenation (ECMO) support was considered the only viable therapeutic option. The patient was therefore transferred to a tertiary referral center with ECMO capability for definitive management.

### 2.3 Persistent instability and escalation to ECMELLA

Upon transfer to the ECMO referral center, invasive hemodynamic assessment with a Swan–Ganz catheter documented a critical and complex profile. The patient was markedly hypermetabolic, with severe hyperpyrexia (core temperature reaching 40.1 °C). Despite Impella support, cardiac index was transiently preserved (2.7 L/min/m^2^), while invasive monitoring showed significant biventricular strain, with elevated central venous pressure (15 mmHg), increased pulmonary artery pressures (38/26 mmHg), and markedly elevated systemic vascular resistance (SVRI 1981 dyn·s/cm^5^/m^2^), consistent with persistent catecholamine-driven vasoconstriction. Importantly, the cardiac index was measured during Impella support and within a transiently compensated interval; therefore, it did not reflect the recurrent rhythm-dependent reductions in effective pump flow or the mismatch between circulatory support and the patient’s extreme metabolic demand. Impella performance became increasingly unreliable during malignant arrhythmias. Recurrent ventricular tachycardia and ventricular fibrillation caused intermittent loss of effective ventricular filling and abrupt reductions in pump flow. Consequently, even with Impella flows approaching 3.0 L/min, isolated LV support could not provide reliable systemic perfusion during the electrical storm or consistently meet the patient’s extreme metabolic demand. The clinical course rapidly progressed to an electrical storm, with repeated episodes of sustained ventricular tachycardia requiring multiple defibrillations, despite aggressive pharmacological therapy including levosimendan, continuous lidocaine infusion, esmolol, diuretics, and vasopressor support with norepinephrine.

**Figure 4:**
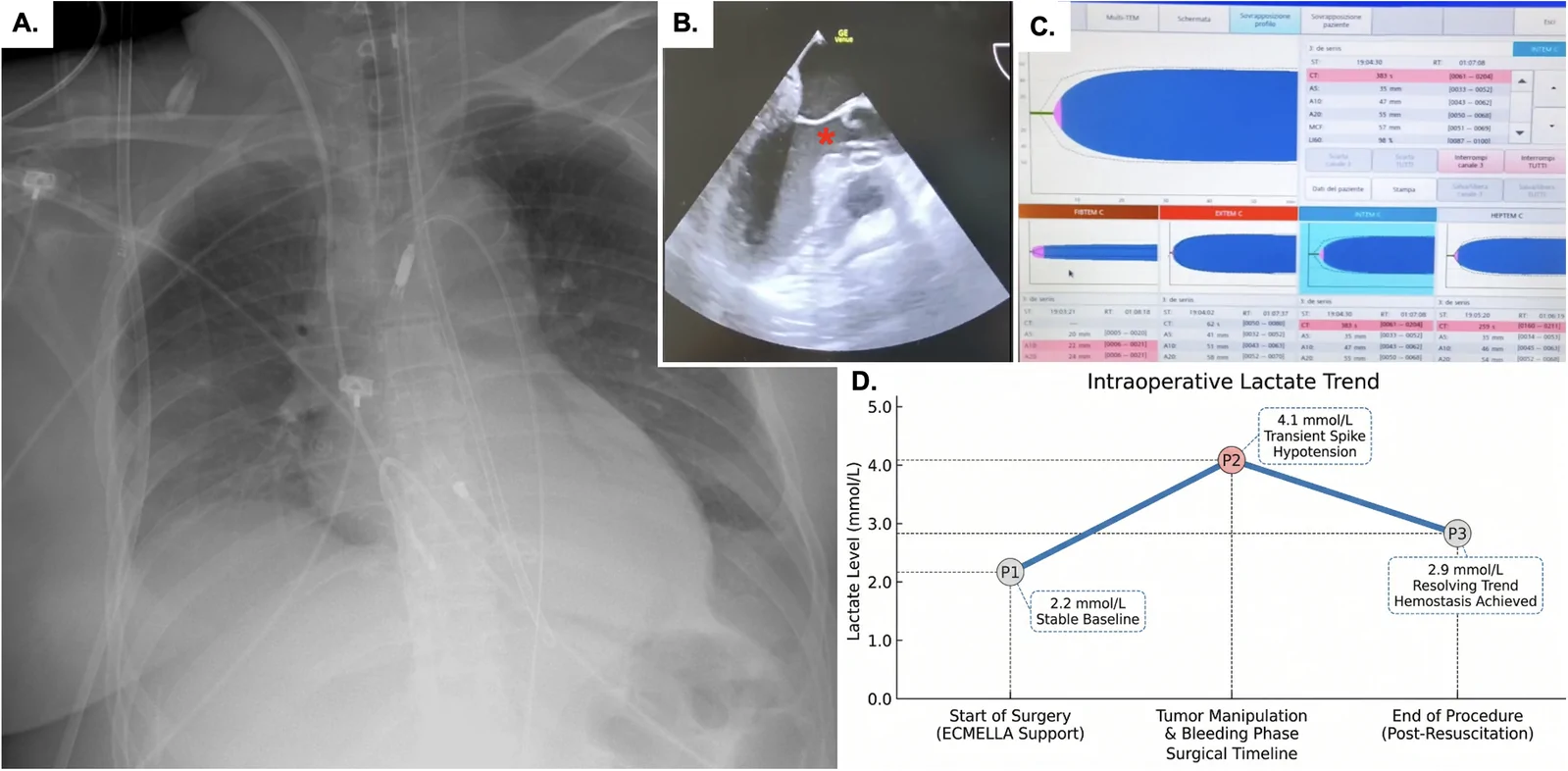
Multimodal monitoring and mechanical support during rescue surgery. **(A)** Chest X-ray of the ECMELLA configuration. Shows the Impella catheter tip properly positioned in the left ventricle (arrowhead) crossing the aortic valve, alongside the peripheral VA-ECMO cannulae. **(B)** Intraoperative Transesophageal Echocardiography (TEE). Mid-esophageal view demonstrating intermittent aortic valve opening (asterisk) despite retrograde ECMO flow, confirming effective LV venting provided by the Impella. **(C)** Rotational Thromboelastometry (ROTEM) Analysis. Intraoperative trace showing preserved clot firmness (MCF), guiding a strategy of hemostatic stability without heparin reversal. **(D)** Intraoperative Lactate Trend. Graph illustrating metabolic status during the surgery. Lactate levels were 2.2 mmol/L at the start under ECMELLA support. A transient spike to 4.1 mmol/L occurred during a hypotensive phase caused by tumor manipulation and mild bleeding, which resolved to 2.9 mmol/L by the end of the procedure following resuscitation.

By that afternoon the patient was in impending cardiovascular collapse. Isolated left ventricular support was no longer sufficient to maintain systemic perfusion during recurrent malignant arrhythmias. VA-ECMO was therefore initiated to provide rhythm-independent systemic perfusion and oxygen delivery, targeting a flow of approximately 3.5 L/min. The Impella CP was maintained at reduced flow (approximately 1.5 L/min) to ensure continuous left ventricular unloading and mitigate the increase in left ventricular afterload associated with peripheral VA-ECMO, thereby establishing an ECMELLA configuration. This combined strategy provided rhythm-independent systemic perfusion together with active LV decompression. Chest radiography confirmed correct positioning of both devices (**Figure 4A**).

At this stage, conventional delayed adrenalectomy after adequate α-adrenergic blockade was considered unattainable. Over the preceding days, repeated attempts at pharmacological α-adrenergic blockade, including intravenous urapidil, had failed to establish a stable therapeutic window. Doses sufficient to attenuate hypertensive surges repeatedly caused profound hypotension despite careful titration and volume support, whereas lower doses were insufficient to prevent recurrent hypertensive episodes. Thus, no durable pharmacological stabilization window could be established.

Although ECMELLA could not eliminate the underlying catecholamine source, it restored systemic perfusion and LV decompression sufficiently to create a temporary, potentially life-saving operative window for definitive source control. In view of the continuing electrical instability, hypermetabolic state, evolving intratumoral heterogeneity on ultrasonography, and progressive organ injury, the multidisciplinary team judged that further delay posed a greater immediate risk than definitive surgery under full mechanical circulatory support.

### 2.4 “Rescue” robotic adrenalectomy

Only a few hours after initiation of the ECMELLA support, emergency robot-assisted complete adrenalectomy of the right adrenal gland was performed during the night between December 4 and 5 under full mechanical circulatory support. This intervention represented a shift from supportive management to definitive source control of the catecholamine-driven crisis. Intraoperative Transesophageal Echocardiography (TEE) confirmed effective LV unloading, with intermittent aortic valve opening despite retrograde ECMO flow, indicating adequate ventricular decompression (**Figure 4B**). Hemodynamic instability during the procedure was managed by dynamic adjustment of ECMO flow, vasopressor titration, and transfusion of packed red blood cells. Coagulation was continuously monitored using rotational thromboelastometry (ROTEM), which showed preserved clot firmness and allowed continuation of the procedure without reversal of systemic anticoagulation (**Figure 4C**). Before tumor manipulation, lactate levels had decreased to 2.2 mmol/L under ECMELLA support, reflecting partial restoration of tissue perfusion. During surgical dissection, a minor vascular injury led to transient hypotension (mean arterial pressure (MAP) <55 mmHg), and a temporary rise in lactate to 4.1 mmol/L. Following tumor removal and hemostasis, hemodynamic conditions improved rapidly and lactate levels decreased to 2.9 mmol/L by the end of the procedure (**Figure 4D**), consistent with reversal of the shock state.

In the immediate postoperative phase (postoperative day 1, POD 1), vascular tone and pulsatility recovered rapidly, with a tendency toward hypertension indicating abrupt withdrawal of catecholamine excess. The Impella device was removed first to reduce vascular complication risk, while VA-ECMO support was progressively weaned and discontinued on POD 5. Post-explant TEE showed persistent severe LV dysfunction (LVEF 25%) but preserved right ventricle (RV) contractility. This preserved RV function represented the key physiological determinant allowing safe weaning from ECMO and bridging of left ventricular stunning with inotropic support (dobutamine 5 µg/kg/min).

### 2.5 Recovery phase and discharge

The patient was extubated on POD 6. Serial echocardiographic assessments showed progressive myocardial recovery. On POD 7, LVEF improved to approximately 30%, although apical ballooning persisted (**Table 2b**). Dobutamine was gradually weaned, and guideline-directed medical therapy with bisoprolol and ramipril was initiated. By POD 9, despite stable hemodynamics, echocardiography revealed dense spontaneous echo contrast in the akinetic apex, indicating high risk of thrombus formation. Full anticoagulation was therefore maintained. The patient was transferred to the Cardiology ward. On POD 14, further improvement in cardiac function was observed, with LVEF reaching approximately 35% (**Table 2b**). After clinical stabilization, the patient was discharged to a specialized cardiac rehabilitation center to continue functional and myocardial recovery. By mid-January, the patient had achieved complete recovery of left ventricular systolic function (**Table 2b**).

### 2.6 Histopathologic features and biochemical follow-up

**Figure 5:**
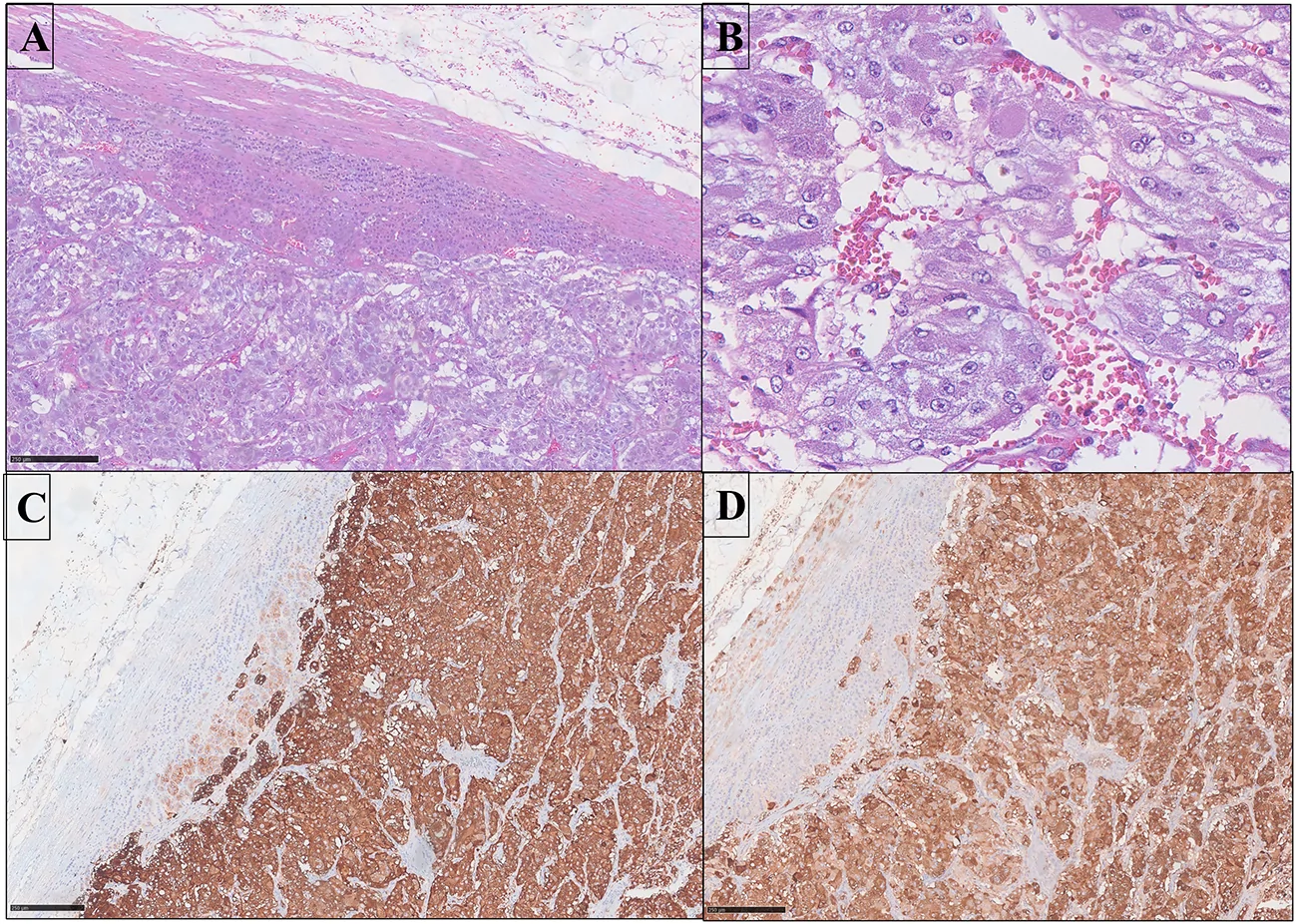
Histological and immunohistochemical features of the adrenal pheochromocytoma: hematoxylin–eosin staining **(A, B)** showing spindle-cell morphology and a solid–trabecular nested growth pattern; immunohistochemical positivity for synaptophysin **(C)** and chromogranin **(D)**.

Histopathological examination of the completely resected right adrenal gland confirmed a pheochromocytoma consisting of nests of tumor cells separated by peripheral capillaries with solid-trabecular growth pattern associated with necrotic and hemorrhagic areas (**Figure 5**). Neoplastic cells resembling normal chromaffin cells, sometimes characterized by spindle-cell morphology, displayed basophilic to amphophilic granular large cytoplasm. Nuclear atypia and cellular pleomorphism were present, but mitoses were very rare. No capsular or vascular invasion was identified, and surgical margins were negative. Immunohistochemistry showed strong positivity for synaptophysin and chromogranin, with negative staining for S-100 and GATA3. The proliferation index (Ki-67) was <1%. According to established scoring systems, the tumor was classified as moderately differentiated (GAPP score 5) with a PASS score of 6, consistent with an intermediate risk profile (6–8). Postoperative serum cortisol, ACTH, and electrolyte levels were within the reference ranges, with no clinical or biochemical evidence of adrenal insufficiency; therefore, glucocorticoid replacement therapy was not required. During cardiac rehabilitation, biochemical reassessment demonstrated normalization of catecholamine excess, with urinary metanephrines within the reference range (**Table 3**), confirming complete biochemical remission after adrenalectomy.

**Table 3:** Post-adrenalectomy hormonal evaluation.

Parameter	24/02/26	Reference
Normetanephrine (µg/24 h)	380.5	<390
Metanephrine (µg/24 h)	104	<320
Homovanillic acid (mg/24 h)	4.5	<6.9
Vanillylmandelic acid (mg/24 h)	4.5	<6.6
5-Hydroxyindoleacetic acid (mg/24 h)	3.6	2-9

## 3 Discussion

Pheochromocytoma and paraganglioma (PPGL) are characterized by marked clinical heterogeneity and do not always present with the classic hyperadrenergic triad or sustained hypertension. A substantial proportion of tumors are detected as adrenal incidentalomas and may be oligosymptomatic at diagnosis (2, 4, 6, 9). Therefore, the absence of overt symptoms does not exclude biologically active disease. Even clinically silent tumors can precipitate catastrophic cardiovascular events when exposed to specific triggers. Pheochromocytoma crisis represents the most severe manifestation, characterized by abrupt catecholamine surge leading to cardiogenic shock, malignant arrhythmias, metabolic acidosis, and multiorgan failure. Catecholamine-induced cardiomyopathy is often reversible and typically presents with Takotsubo or reverse-Takotsubo patterns, reflecting myocardial stunning rather than coronary obstruction (1, 10). In the present case, the crisis followed high-dose glucocorticoids administered for contrast allergy prophylaxis. Although multiple contributing factors may have been present, glucocorticoids likely played a central triggering role (2, 6, 9). This observation is consistent with previous reports showing that exogenous glucocorticoids can precipitate pheochromocytoma crisis, including severe multisystem presentations and fatal outcomes (11). The mechanisms are not fully understood but may involve increased catecholamine synthesis and release, as well as altered tumor handling of catecholamines. Importantly, crisis has been described not only after therapeutic glucocorticoid administration but also after dexamethasone suppression testing, particularly at higher cumulative doses (11).

These findings have direct implications for the management of adrenal incidentaloma. Current guidelines recommend biochemical exclusion of pheochromocytoma and performance of a 1-mg dexamethasone suppression test (DST) as part of routine evaluation (4). Although low-dose DST is generally considered safe, published reports indicate that dexamethasone, even at modest doses, may trigger crisis in susceptible patients (11). In addition, patients with adrenal masses are often exposed to substantially higher glucocorticoid doses during contrast allergy premedication. Our case emphasizes that in patients with indeterminate adrenal lesions, particularly those with features suggestive of higher malignancy risk (e.g., size ≥4 cm and/or unenhanced attenuation >20 HU, and/or low delayed contrast media washout), early biochemical exclusion of pheochromocytoma should be prioritized before any glucocorticoid exposure, including contrast premedication protocols. This approach is not only diagnostically appropriate but may be critical for patient safety (4, 11).

Catecholamine-induced cardiomyopathy is a potentially reversible cause of severe cardiogenic shock (1, 10). In this context, temporary mechanical circulatory support (tMCS) plays a central role: VA-ECMO has been shown to stabilize refractory shock and bridge patients to recovery once catecholamine excess is controlled (3, 10, 12). Impella provides effective LV unloading and may reduce myocardial stress. However, its limitations are evident in the presence of arrhythmic instability and extreme metabolic demand. In our patient, recurrent ventricular arrhythmias impaired preload and intermittently reduced pump performance. Moreover, even maximal Impella support was insufficient to match the extreme hypermetabolic state associated with severe hyperpyrexia (core temperature 40.1 °C).

Escalation to combined VA-ECMO and Impella support (ECMELLA) provided a more complete physiological solution. VA-ECMO ensured systemic perfusion independent of cardiac rhythm, while Impella maintained LV unloading and counteracted ECMO-induced afterload increase (13, 14). This complementary effect has been demonstrated in cardiogenic shock populations and supported by combined-support analyses (13–15). In our case, intraoperative TEE confirmed effective LV decompression under dual support. Additionally, the ECMO circuit allowed rapid control of hyperpyrexia, contributing to metabolic stabilization. Most reported cases describe ECMO or ECMELLA as a bridge to myocardial recovery followed by delayed adrenalectomy (3, 16–18). Persistent instability despite Impella prompted escalation to ECMELLA, which was then used as a bridge to immediate tumor removal rather than to cardiac recovery. To our knowledge, this represents one of the first reports of ultra-early adrenalectomy performed under full ECMELLA support in the absence of hemodynamic stabilization.

Current recommendations favor medical stabilization and adequate α-adrenergic blockade before adrenalectomy, because emergency resection has historically been associated with greater perioperative morbidity and mortality (19, 20). However, evidence specifically addressing the optimal timing of surgery in pheochromocytoma crisis remains limited.

While delayed surgery remains appropriate whenever durable stabilization can be achieved, this strategy may not be feasible in the setting of refractory crisis and multiorgan dysfunction (3, 16, 21). In such cases, the tumor itself is the primary driver of hemodynamic collapse, and definitive source control becomes the only effective therapy. In our patient, α-adrenergic blockade could not be titrated to establish a stable therapeutic window because hypertensive surges alternated with profound hypotension, while malignant arrhythmias and hypermetabolic instability persisted. Moreover, the evolving intratumoral heterogeneity observed on ultrasonography, subsequently corroborated by necrotic and hemorrhagic areas at histopathological examination, raised concern for an unstable tumor source potentially contributing to episodic catecholamine release.

ECMELLA restored systemic perfusion and LV decompression sufficiently to create a temporary operative window but could not eliminate the underlying catecholamine source. Complete right adrenalectomy was therefore undertaken as a rescue intervention after pharmacological stabilization and isolated LV support had failed. Following tumor removal, the rapid recovery of vascular tone and native pulsatility, together with subsequent myocardial recovery, was temporally consistent with interruption of catecholamine exposure (3). Although causality cannot be established from a single case, this interpretation is supported by the well-recognized reversibility of catecholamine-induced cardiomyopathy following biochemical cure (10).

Decisions regarding VA-ECMO weaning should not rely solely on LVEF but rather on a multiparametric assessment including hemodynamic stability during progressive flow reduction, native arterial pulsatility, end-organ perfusion, metabolic recovery, and biventricular function (22, 23). In our patient, VA-ECMO was discontinued despite persistent severe LV systolic dysfunction because native pulsatility and vascular tone had recovered, right ventricular function was preserved, and systemic perfusion remained adequate with inotropic support. Although LVEF remained approximately 25%, the LV dysfunction was considered potentially reversible catecholamine-induced myocardial stunning and was bridged with dobutamine until further recovery (10, 12).

## 4 Conclusion

Pheochromocytoma crisis can occur abruptly even in previously paucisymptomatic patients and may be precipitated by commonly used drugs, including glucocorticoids. This case highlights the importance of biochemically excluding pheochromocytoma before glucocorticoid exposure in patients with indeterminate adrenal masses. Although delayed adrenalectomy after appropriate pharmacological stabilization remains the preferred strategy whenever achievable, in highly selected refractory cases complicated by cardiogenic shock, malignant arrhythmias, and failure to achieve durable stabilization, combined mechanical circulatory support may provide a bridge to life-saving definitive surgical treatment. The glucocorticoid-catecholamine axis represents an underrecognized and potentially preventable trigger of pheochromocytoma crisis; its recognition in the diagnostic workup of adrenal incidentalomas may prevent catastrophic cardiovascular events.

## Data Availability

The original contributions presented in the study are included in the article/supplementary material. Further inquiries can be directed to the corresponding authors.
